# Canine sense of quantity: evidence for numerical ratio-dependent activation in parietotemporal cortex

**DOI:** 10.1098/rsbl.2019.0666

**Published:** 2019-12-18

**Authors:** Lauren S. Aulet, Veronica C. Chiu, Ashley Prichard, Mark Spivak, Stella F. Lourenco, Gregory S. Berns

**Affiliations:** 1 Department of Psychology, Emory University, Atlanta, GA 30322, USA; 2 Comprehensive Pet Therapy, Atlanta, GA 30328, USA

**Keywords:** approximate number system, canine cognition, quantity discrimination, fMRI, parietal cortex

## Abstract

The approximate number system (ANS), which supports the rapid estimation of quantity, emerges early in human development and is widespread across species. Neural evidence from both human and non-human primates suggests the parietal cortex as a primary locus of numerical estimation, but it is unclear whether the numerical competencies observed across non-primate species are subserved by similar neural mechanisms. Moreover, because studies with non-human animals typically involve extensive training, little is known about the spontaneous numerical capacities of non-human animals. To address these questions, we examined the neural underpinnings of number perception using awake canine functional magnetic resonance imaging. Dogs passively viewed dot arrays that varied in ratio and, critically, received no task-relevant training or exposure prior to testing. We found evidence of ratio-dependent activation, which is a key feature of the ANS, in canine parietotemporal cortex in the majority of dogs tested. This finding is suggestive of a neural mechanism for quantity perception that has been conserved across mammalian evolution.

## 1. Introduction

Whether avoiding predators or foraging for food, it is evolutionarily imperative that animals perceive and represent visual information regarding quantity. Extensive behavioural evidence suggests that non-human animals share with humans a sense of numerosity—that is, sensitivity to numerical information that does not rely on symbolic thought or education [1,2]. This approximate number system (ANS), a system for rapidly assessing the approximate number of items present in an array, appears to be present both across the animal kingdom, and early in human development, with even newborn infants possessing the remarkable ability to discriminate stimuli based on numerosity [3].

Although many animals—including monkeys [4], fish [5], bees [6] and dogs [7,8]—display behavioural sensitivity to numerosity, it is unclear whether the neural mechanism that underlies this ability is conserved across species. In human and non-human primates, evidence suggests that parietal cortex is the primary locus of this capacity [9,10], though other cortical regions have also been implicated [11,12]. However, neural evidence for numerical abilities in non-human primates typically has required the animal to complete extensive training and to make explicit number judgements [7,10]. Consequently, it is unclear what mechanisms underlie the capacities that these animals exhibit spontaneously [13,14].

Assessment of spontaneous numerical abilities in non-human animals typically necessitates the use of biologically relevant stimuli, such as food or mates, in order to sufficiently motivate the animal to engage in a numerical choice. As a result, when animals fail to discriminate on the basis of numerosity in these paradigms, it is difficult to determine whether this reflects a lack of numerical abilities, or rather, a lack of motivation for the stimuli used (for further discussion of this issue, see [15]).

Circumventing several of these concerns, recent work has found evidence of spontaneous neural encoding of numerical information, localized in the endbrain of crows [16], a region considered analogous to the prefrontal cortex (PFC) in primates [17]. In this work, crows did not receive training on numerical discrimination, nor did they make explicit numerosity judgements. They completed an orthogonal task (i.e. colour judgement) on non-biologically relevant stimuli (i.e. dot arrays). However, given the early divergence of crows from mammals in phylogenetic history, it is uncertain to what extent the neural mechanisms observed in crows are shared with mammals, especially given the suggestion that the endbrain is mostly homologous with the PFC, which is a region less frequently implicated in spontaneous number perception in humans. By contrast, if spontaneous number perception were subserved by parietal mechanisms in non-primates, this commonality would confirm a deeply conserved neural mechanism of non-symbolic number representation.

To address this question, we used awake canine functional magnetic resonance imaging (fMRI) to assess sensitivity to visual numerosity in pet dogs. Because this methodology allows assessment of number representations in the absence of number-specific training, and without relying on behavioural responses, we avoided common weaknesses in comparative numerical cognition. In the present study, dogs passively viewed dot arrays that varied in numerical value.

Following previous work [18–20], we predicted that if dogs, like human and non-human primates, have a dedicated region of the cortex for representing non-symbolic numerical quantity, then activation in this region should increase as the ratio between alternating dot arrays increases. That is, a number-sensitive region of cortex will exhibit greater activation when the numerical values of the stimuli are more dissimilar (e.g. 2 versus 10 dots) than when numerical values are constant (e.g. 6 versus 6 dots), despite constant cumulative surface area and variable element size, consistent with Weber's Law [21–23].

Owing to limitations on the duration of scanning sessions with awake dogs [24,25], our paradigm was different from the common ‘oddball’ paradigm used with humans [18], in which a numerical value is adapted to over many trials and then probed with an oddball value. Recent behavioural [26] and neural [20] findings suggest a long adaptation period is not necessary for demonstrating ratio effects. Thus, we used a block design in which we predicted a parametric increase in activation as a function of the increase in block ratio.

## 2. Material and methods

### (a) Participants

Eleven awake, unrestrained dogs (table 1), were scanned in a Siemens 3T Trio MRI scanner (electronic supplementary material, video S1). Prior to testing, all dogs completed a training programme to be desensitized to the scanner environment through behaviour shaping and positive reinforcement [25]. All dogs had previously participated in fMRI studies while viewing stimuli on a projection screen but had no prior training on numerical discrimination.

**Table 1.** Dogs’ demographic information.

dog	breed	sex	age (years)
Bhubo	Boxer mix	M	2
Caylin	Border Collie	F	10
Daisy	Pitbull mix	F	10
Eddie	Labrador Golden mix	M	7
Kady	Labrador	F	8
Koda	Pitbull mix	F	3
Libby	Pitbull mix	F	13
Pearl	Golden retriever	F	8
Tallulah	Carolina dog	F	6
Truffles	Pointer mix	F	13
Zen	Labrador Golden mix	M	9

### (b) Stimuli

Stimuli were 75 dot arrays comprising light grey dots on a black background (800 × 800 pixels). For each numerosity used (2, 4, 6, 8 and 10), stimuli varied in cumulative area (i.e. the total grey on each image). For each numerosity, cumulative area was 10, 20 or 30% of the total stimulus. For each numerosity, 15 unique stimuli were used (five stimuli per cumulative area value). In each stimulus, individual dot size varied up to 30%. Dot location varied randomly. Critically, these controls minimize the influence of non-numerical properties, in order to ensure that the results can be attributed to changes in numerical value [27,28]. In accordance with current estimates of canine visual acuity (approx. 20/75; [29]), all stimuli were analysed to ensure that the inter-dot distances were large enough for dogs to individuate.

### (c) Block design

**Figure 1.**
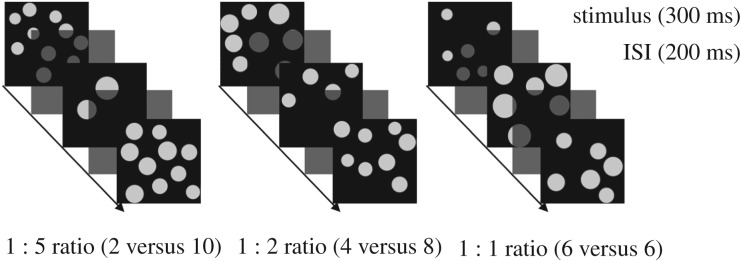
Block design of the present study. Dogs were presented with alternating stimuli of 2 and 10 (1 : 5 ratio), 4 and 8 (1 : 2 ratio) or 6 and 6 (1 : 1 ratio) dots in a block fMRI design.

During scanning, dogs passively viewed dot array stimuli presented on a screen placed in the rear of the scanner. Dogs were presented with alternating stimuli of 2 and 10 (1 : 5 ratio), 4 and 8 (1 : 2 ratio) or 6 and 6 (1 : 1 ratio) dots in a block fMRI design (figure 1). Stimuli were presented using PsychoPy software [30]. Each block contained 20 stimuli and lasted 10 s (electronic supplementary material, videos S2 and S3). An experimenter standing in the rear of the scanner manually initiated each block to ensure that dogs were still in a suitable position within the scanner. Block onset times were recorded using an MRI-compatible button box. The inter-block intervals lasted approximately 10 s. Dogs were provided a food reward by their owner randomly throughout each run (always during the inter-block interval). Each run contained 20 blocks (randomized) and lasted approximately 5 min.

### (d) MRI scan acquisition

Dog training and fMRI protocol were consistent with the procedures previously used in awake dog fMRI studies [24,25]. The scans were obtained using a Siemens 3T Trio MRI scanner. To obtain functional scans, a single-shot echo-planar imaging sequence was used to acquire volumes of 22 sequential 2.5 mm slices with a 20% gap (TE = 25 ms, TR = 1260 ms, flip angle = 70°, 64 × 64 matrix, 2.5 mm in-plane voxel size, FOV = 192 mm). For each individual, approximately 1300–2000 functional volumes were acquired over the course of two to five runs. For each dog, the total scan session lasted for a maximum of 40 min.

### (e) Preprocessing

AFNI (NIH) was used for both preprocessing and statistical analysis. Preprocessing of the fMRI data included motion correction, censoring and normalization using AFNI and its associated functions. Two-pass, six-parameter rigid-body motion correction was used based on a hand-selected reference volume for each dog that corresponded to the most representative position of the dog's head within the magnet bore across runs. Aggressive censoring removed questionable volumes from the fMRI time sequence because dogs can move between trials and when consuming rewards. Censoring was performed with respect to both signal intensity and motion, in which volumes with more than 1 mm of scan-to-scan movement were flagged as spurious and censored from further analysis. Smoothing, normalization and motion correction parameters were identical to those described in previous studies [31]. The Advanced Normalization Tools software [32] was used to spatially normalize the mean of the motion-corrected functional images to the individual dog's structural image. To improve signal-to-noise ratio, the data were then spatially smoothed with a 4 mm Gaussian kernel.

### (f) Region of interest analysis

Each dog served as its own control for cross-validation as we performed a fourfold split on the data. Seventy-five per cent of the data was used to localize the most likely number-selective region of interest (ROI) in each dog, while the remaining 25% was held out for independent validation. Because of the variability in response threshold across dogs, we used a customized approach that identified the most likely cluster of voxels correlated with number ratio in each dog. This was done by varying the voxel threshold of the statistical map for each dog so that one or two clusters remained that were 10–40 voxels in extent (electronic supplementary material, table S1). Once identified, the independent estimate from the holdout data was extracted from this ROI and submitted to a *t*-test across dogs.

For each dog, a general linear model was estimated for each voxel using the 3dDeconvolve function in AFNI. Non-task regressors included the six motion regressors obtained from motion correction. Because neural activation was predicted to vary linearly with ratio, an amplitude-modulated response model was used in which a parametric modulator was assigned to each block based on the numerical ratio of the block (1, 2 or 5). This yielded two columns: the main effect of dot stimuli and one modulated by the ratio of dots in a block. To allow for independent localization and test data, we further partitioned the design matrix into separate columns for either localization (75% of blocks) or testing (25% of blocks). We ensured that each test set had at least two instances of each condition represented in each run. To minimize any effect of fatigue or familiarization, we ensured that average onset times were matched (i.e. did not differ by more than 5 s) across localization and test sets.

## 3. Results

**Figure 2.**
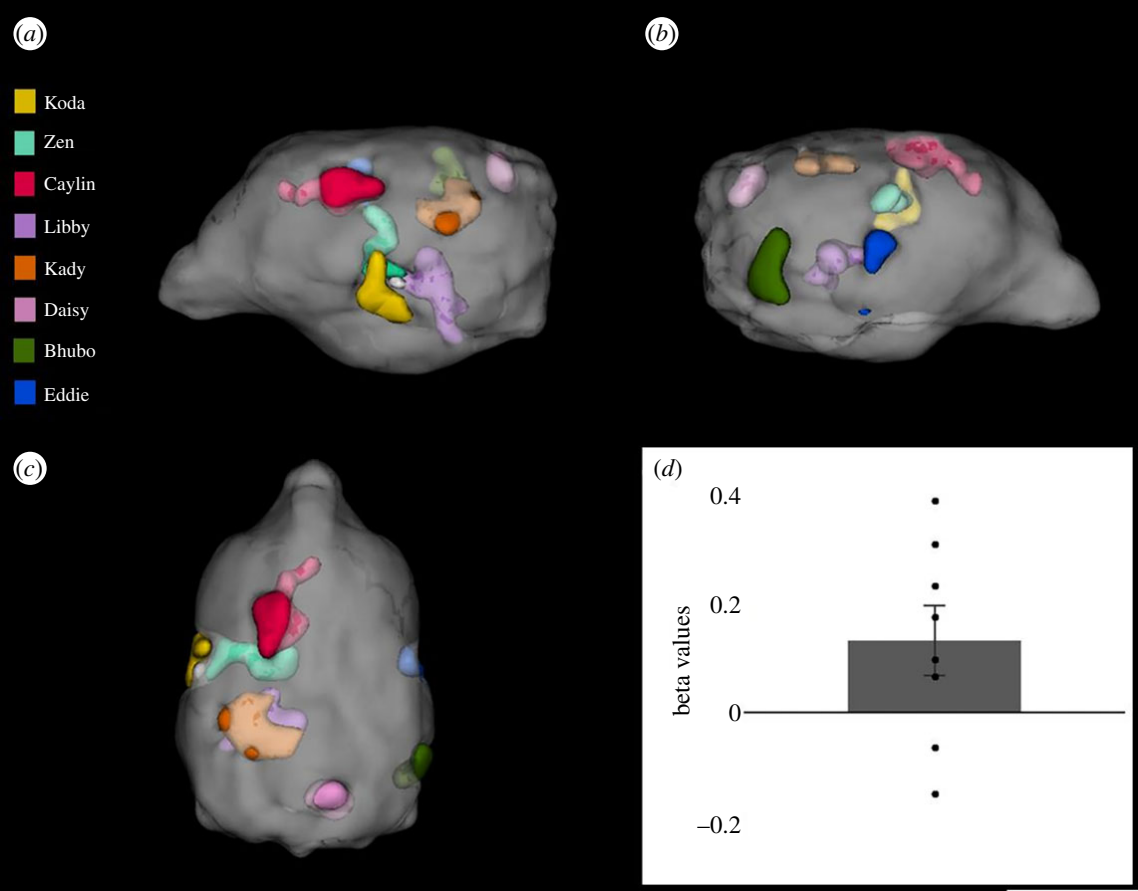
Location of number-sensitive regions for all dogs and effect size in held-out data. For visualization and comparison of location, the regions of interest (ROIs) have been spatially normalized and overlaid on a high-resolution dog brain atlas [33]. Each colour represents the ROI of one dog. (*a*) Left-sided view. (*b*) Right-sided view. (*c*) Dorsal view (nose at top). Of the 11 dogs scanned, three did not exhibit ratio-dependent activation: Pearl, Tallulah and Truffles (8 and 13 years old, respectively). Of the eight dogs that did exhibit ratio-dependent activation, there was no correlation between age and ROI size or beta value (*p* values > 0.826). (*d*) Beta values from the number-sensitive ROIs in the held-out data for block ratio as a predictor of activation. Data points represent individual dogs. Error-bar shows s.e.m. (*t*_7_ = 2.01, *p* = 0.042, one-sided).

In eight of 11 dogs (electronic supplementary material, table S1), we identified regions of cortex that exhibited increasing activation with numerical ratio in parietotemporal lobes according to a high-resolution canine brain atlas ([33]; figure 2*a–c*). Although there was variability in the specific location of each dog's ROI, this is perhaps unsurprising given the different breeds in the current study [34].

Crucially, to assess whether dogs, like primates, have number-sensitive regions of cortex, we examined whether activation in the localized ROIs was ratio-dependent in the held-out data. We found that block ratio was significantly correlated with the level of activation in these regions (*t*_7_ = 2.01, *p* = 0.042, one-sided; figure 2*d*), consistent with a ratio-dependent effect. These findings suggest that dogs have a visual sense of number subserved by similar parietotemporal mechanisms to those in primates [10,11].

## 4. Discussion

In summary, we examined the neural underpinnings of the ANS in dogs and found evidence of activation in parietotemporal regions that varied as a function of numerical ratio, akin to that previously observed in primates. Given that number-specific training was not required, this research provides novel evidence that dogs spontaneously discriminate visual numerosity even when arrays are equated for cumulative area while remaining variable in individual element size. Although evidence from even a single animal provides an important proof of concept for this ability, the work here shows that the majority of dogs demonstrated spontaneous ratio-dependent neural activation, providing greater generalizability and stronger support for evolutionarily conserved neural mechanisms. There are still open questions about dogs' ability to discriminate arrays based on other ensemble properties, such as average element size, which was variable in the current study and may have been discriminable. In naturalistic settings, however, such information is highly correlated with numerosity and likely to be used in combination with numerosity [35].

Previous work, in both human and non-human animals, suggests separate systems for representing ‘small’ (i.e. 1–3) and ‘large’ (i.e. greater than 4) numerosities [36]. A common view is that small numbers are represented by an object file system that represents exact numerosity and does not exhibit ratio-dependence. Here, dogs exhibited ratio-dependent activation for numerical values that included small and large numerosities (see also [37,38]). Although this could suggest that non-human animals possess a single system for representing both small and large numerical values [39,40], ratio-dependence for small numbers could also reflect the particular task demands, namely speeded presentation of stimuli, which better equates the processing demands for different numerosities [41].

Consistent with recent evidence for emergent numerical capacities in computational models of object recognition [42], the present work provides evidence for neural representations of visual quantity in the absence of explicit training. It also suggests that, like in humans, number perception occurs in parietotemporal cortex in the dog, a non-primate mammal. Taken together, our findings suggest that the ability to represent numerosity and the mechanisms supporting this system are deeply conserved over evolutionary time, perhaps owing to a role in foraging or predation, and persists in a domesticated species.

## Supplementary Material

Supplemental Information

## Supplementary Material

Video 1

## Supplementary Material

Video 2

## Supplementary Material

Video 3

## Data accessibility

The dataset supporting this article are available from the Dryad Digital Repository: https://doi.org/10.5061/dryad.02ts21t [43].
