# Supplementary material for: Canine sense of quantity: evidence for numerical ratio-dependent activation in parietotemporal cortex
Source: Biol Lett. 2019 Dec 18;15(12):20190666. doi: 10.1098/rsbl.2019.0666 (PMC6936025; doi:10.1098/rsbl.2019.0666)
Supplement: Supplemental Information [file rsbl20190666supp1.docx]

**Supplemental Information**

Canine sense of quantity: evidence for numerical ratio-dependent activation in parietotemporal cortex

Lauren S. Aulet, Veronica C. Chiu, Ashley Prichard, Mark Spivak, Stella F. Lourenco & Gregory S. Berns

**Supplemental Table 1. Number region size and threshold significance**

| **Dog** | **Voxels (*N*)** | ***p*** |
| --- | --- | --- |
| Bhubo | 20 | 0.05 |
| Caylin | 38 | 0.01 |
| Daisy | 13 | 0.05 |
| Eddie | 27 | 0.02 |
| Kady | 22 | 0.001 |
| Koda | 24 | 0.05 |
| Libby | 21 | 0.09 |
| Zen | 20 | 0.05 |

Dogs’ number (ratio-dependent) ROI voxel size and *p* value for thresholds.

**Supplemental Video 1.** Callie entering head coil in scanner.

**Supplemental Video 2**. Callie watching dots. Front view (not scanning).

**Supplemental Video 3**. Bhubo watching dots. Rear view (during scanning).
